# Supplementary material for: A survival analysis based volatility and sparsity modeling network for student dropout prediction
Source: PLoS One. 2022 May 5;17(5):e0267138. doi: 10.1371/journal.pone.0267138 (PMC9071151; doi:10.1371/journal.pone.0267138)
Supplement: S1 File — (DOCX) [file pone.0267138.s001.docx]

A Survival Analysis based Volatility and Sparsity Modeling Network for Student Dropout Prediction

Feng Pan, Bingyao Huang, Zhenyu Wu, Yang Ji, Xinning Zhu, Chunhong Zhang, Zhanfei Ma & Zhengchen Li

Corresponding Author: Yang Ji

Email: jiyang@bupt.edu.cn

This PDF file Includes

**Supporting Text**

[Definition of Survival analysis 2](#_Toc87372046)

[Detailed Derivation of the likelihood function for a sample $\boldsymbol{i}$ 3](#_Toc87372047)

[Supporting Dataset 3](#_Toc87372048)

**Supporting Dataset**

**References for SI Citations**

Definition of Survival analysis

| *t* | The current timestamp. |
| --- | --- |
| $T^{i}$ | The duration time of sample *i*. |
| *E* | The dropout event. |
| *N* | The total number of students registered in a dataset. |
| $f\left( t \right)$ | A probability density function. |
| $F\left( t \right)$ | A cumulative distribution function. |
| $S\left( t \right)$ | A survival function. |
| $\lambda\left( t \right)$ | A hazard function. |
| Table S1. Summary of notation used in construction of the survival analysis model. | |

Survival analysis models one or more non-negative random variables (i.e. survival time $T$) until the occurrence of an interest event. It has two fundamental functions to characterize the non-negative survival time $T$. One is the survival function$S\left( t \right)$, which indicates the probability of the event having not occurred by time $t$:

$$S\left( t \right)=P\left( T\geq t \right)=1-F\left( t \right)=\int_{t}^{\infty} f(x)dx$$

The other is the hazard function $\lambda\left( t \right)$, which represents the instantaneous hazard rate at time $t$ given no event occurred before time $t$ :

$$\lambda\left( t \right)=\lim_{dt\to0} \frac{P\left\{ t\leq T<t+dt \right| T\geq t\}}{dt}=\frac{f(t)}{S(t)}$$

Obviously, $\lambda\left( t \right)=-\acute{S(t)}/S(t)$ and there is:

$$S\left( t \right)=e^{-\int_{0}^{t} \lambda(x)dx}$$

If the observation time $T$ is a discrete variable(seconds, minutes or days), the correlation between the survival function and the hazard function is:

$$S_{t}=e^{-\sum_{k=1}^{t} \lambda_{k}}$$

Detailed Derivation of the likelihood function for a sample $\boldsymbol{i}$

The likelihood function for a sample $i$ is the product of the probability of an event having occurred before $T^{i}$ and the probability of an event not having occurred by $T^{i}$:

$${P\left\{ T<T^{i} \right\}}^{y^{i}}\cdot{P\left\{ T\geq T^{i} \right\}}^{1-y^{i}}$$

$$=\left( F\left( T^{i} \right) \right)^{y^{i}}\cdot{S\left( T^{i} \right)}^{1-y^{i}}$$

$$=\left( 1-e^{-\sum_{t=1}^{T^{i}} \lambda_{t}} \right)^{y^{i}}\cdot\left( e^{-\sum_{t=1}^{T^{i}} \lambda_{t}} \right)^{1-y^{i}}$$

$$=\left( e^{\sum_{t=1}^{T^{i}} \lambda_{t}}-1 \right)^{y^{i}}\cdot e^{-\sum_{t=1}^{T^{i}} \lambda_{t}}$$

Supporting Dataset

KDDCup 2015 dataset is available at <https://www.biendata.xyz/competition/kddcup2015/data/>.

XuetangX dataset is available at <http://moocdata.cn/data/user-activity>.

The minimal data set underlying the results described in our manuscript is available in the Zenodo repository: <https://doi.org/10.5281/zenodo.5914059>.

Supplementary References

1. Zheng P, Yuan S, Wu X. SAFE: A Neural Survival Analysis Model for Fraud Early Detection. Proceedings of the {AAAI} Conference on Artificial Intelligence. 2019;33(01):1278-1285.
