# Supplementary material for: A survival analysis based volatility and sparsity modeling network for student dropout prediction
Source: PLoS One. 2022 May 5;17(5):e0267138. doi: 10.1371/journal.pone.0267138 (PMC9071151; doi:10.1371/journal.pone.0267138)
Supplement: Attachment — Submitted filename: Response to Reviewers.docx [file pone.0267138.s002.docx]

Dear Editor and Reviewers,

Thank you for providing three reviewers on paper “A Survival Analysis based Volatility and Sparsity Modeling Network for Student Dropout Prediction” (Manuscript PONE-D-21-35650). We appreciate the reviewers’ and editor’s comments very much to improve the quality of this paper. The revision has been completed by replying point by point to the comments in the rebuttal letter. All major changes are highlighted in red color in the revised manuscript.

We are looking forward to hearing back from you soon.

Sincerely,

Feng Pan

Beijing University of Posts and Telecommunications

(On behalf of all co-authors)

*****************************************************************************

**Response to reviewers’ review**

- ***Reviewer: 1***

*Comments for the authors:*

1. *" Research questions, that drive the paper, should be built in the introduction from an ongoing and pertinent bibliography (up to 2021). These should be of global interest and not focused to a particular local problem. Identifying a research gap is not enough; key is showing its significance to the field. "*

**[Authors’ response]:** Thank you very much for for pointing out this important problem. We have re-written the introduction part according to the Reviewer’s suggestion. The relevant contents are provided below as a screen dump for your quick reference. We hope the correction will meet with approval.

2. *" Answer your research question in the conclusions; what did we learn compared with current, significant research . "*

**[Authors’ response]:** Thank you very much for pointing out this important problem. As Reviewer 1 suggested that, we have re-written the conclusion part according to the Reviewer’s comments. The relevant contents are provided below as a screen dump for your quick reference. To be specific, we believe the statement of "In this paper, we propose a novel end-to-end framework named SAVSNet for student dropout prediction, which focuses on addressing the problems of data volatility and sparsity.”on line 630-632, and the statement of "The experiment results on two real-world datasets validate that the treatment of volatility and sparsity improves predictive performance. " on line 639-641, have answered our research question and revealed what did we learn comparaed with current, significant research.

3. *" How general are your results? These have to be of interest to the whole community. Relate these with your limitations. "*

**[Authors’ response]:** Thank you very much for pointing out this important problem. We have followed your suggestions to emphasize the generalization of our results in the discussion section. The main corrections in the manuscript are as flowing:

"It demonstrates that our designed modules can effectively deal with the issue of data volatility and sparsity, and the techniques proposed can be generalized to any volatile and sparse sequence scenarios. "

In addition, we have related the generalization with our limitations in the discussion section. The relevant contents are provided below as a screen dump for your quick reference.

4. *" In the conclusion section, the limitations of this study, suggested improvements of this work and future directions should be highlighted. "*

**[Authors’ response]:** Thanks for your great suggestion on improving the accessibility of our manuscript. We have highlighted the limitations of this study, suggested improvements of this work and future directions in the conclusion section. The relevant contents are provided below as a screen dump for your quick reference.

5. *" The research problem is not clear, and it needs to be supported and explained why the researchers did the research. "*

**[Authors’ response]:** Thank you very much for pointing this out. We have followed your suggestions to summarize our research problem in the introduction section. The relevant contents are provided below as a screen dump for your quick reference.

At last, the authors would like to give many thanks to Reviewer 1’s constructive comments for improving the quality of this paper.

******************************************************************

- *Reviewer: 2*

*Comments for the authors:*

1. *" Title. The authors study the dropout rate of online learning courses, so we should make it clear, otherwise it will be easily confused with traditional dropout rate. "*

**[Authors’ response]:** We are very sorry for the confusion caused by our unclear expression. We have added the description on the difference between online and traditional dropout rate in the introduction section, which is as follows:

“The spread of Covid-19 has brought new opportunities for e-learning, especially for the online education platforms represented by Massive Open Online Courses (MOOCs), which attract people’s attention once again. However, due to the scarcity of adequate supervision and necessary restrictions for e-learners, online students have a much higher chance of dropping out than those attending conventional classrooms [1].”

2. *" Abstract. Authors should focus their writing on the content of this study instead of spending a lot of time explaining the research reasons. "*

**[Authors’ response]:** Thank you very much for pointing out this important problem. We have re-written the abstract part according to the Reviewer’s suggestion. The presentations of the research reasons have been condensed. The relevant contents are provided below as a screen dump for your quick reference. We hope it can achieve your request.

3. *" Introduce. I think the authors should introduce the previous literature analysis in this research field, which can help readers to understand more intuitively why this research is meaningful, rather than the authors' independent explanation. In addition, how is Survival Analysis adopted as an effective method to solve this problem? For a simple example, Survival Analysis has been applied to similar fields and is considered effective. I think the authors can briefly explain it from this angle. The significance of this research is actually more credible than that of self-explanation. In the last paragraph of this part, this is the first time i saw this kind of structure introduction about the full text in the manuscript, and I suggest that the authors adopt experimental design or process to explain it. "*

**[Authors’ response]:** Thanks very much for your comments, which are very helpful to improve the quality of this article. We have followed your suggestions to re-write all the Introduction part in the manuscript. We have strengthened the analysis of previous literature and removed some individual independent explanation. Moreover, we have removed the introduction about the full text in the last paragraph of this part. The relevant contents are provided below as a screen dump for your quick reference and we hope it can meet your request.

4. *" Related Works. I don't quite understand this expression adopted by the authors, which is consistent with the suggestions I put forward in my introduction. I think this part is more like a theoretical basis or a literature review. Specific authors can refer to the specific requirements of journals for revising the manuscript framework. At the same time, in this part, I only saw the introduction of three key words, but I didn't see in detail the collation and summary of the authors' previous research on these contents. I think this is very important, because it can highlight the importance and value of this research. "*

**[Authors’ response]:** Thank you very much for pointing out this important problem. We have added the collation and summary of the Related Works. The description is as follows:

“In this section, some classical methods of student dropout prediction are introduced first. Then, we list the survival analysis methods proposed so far. Finally, we review two lines of work relevant to ours, namely volatility and sparsity modeling.”

5. *" Methods. I think that the authors' calculation of the model is OK, but what needs to be pointed out is that they are relatively weak in literal expression, which makes my reading of the whole calculation process not smooth. I suggest that the authors further sort out the logic of expression. "*

**[Authors’ response]:** Thank you very much for your positive evaluation, we have gone through the whole manuscript in the methods section and revised it according to your important suggestions. The specific manuscript will not be pasted here as it is too voluminous, and details can be found in the revised version.

6. *" Experiment. First of all, I need a serious description of this part, which means that it needs to be greatly revised. The authors are not clear about the source of the data. It is not advisable to introduce only some numerical values. Every step of the experiment needs to be explained clearly. At the same time, I realize that if it involves the examination of courses and students, whether this research has obtained the ethical review is very important for publishing, and it is the criterion that authors need to follow. If it is not involved, it needs to be clearly explained. "*

**[Authors’ response]:** Thank you very much for your comments. We have re-written the Dataset Description part and described the source of the data. Furthermore, we have highlighted in the manuscript that both the datasets we used have been anonymized and ethically reviewed. The relevant contents are provided as a screen dump for your quick reference.

7. *" Result. I actually agree with the authors' statements in the results section, but I still want to suggest whether the authors should incorporate other proven methods to further verify the accuracy of your research, because I personally don't think the existing sensitive analysis is enough to verify the reliability of the research. "*

**[Authors’ response]:** Thank you very much for pointing out this important problem. We have followed your suggestions to revise the Sensitive Study. It has been noted that the sensitive analysis approach is a common technique used to compare different versions of the same factor and is seen as a critical component of internal reliability.The relevant contents are provided below as a screen dump for your quick reference.

8. *" Conclusion. Maybe it's because of the space. I don't think the authors have done a good job in this part. I suggest that the authors elaborate on some suggestions of this study for reducing the dropout rate. That is, integrating and analyzing the findings of the existing research, the reality and the findings of this study will help to study the by going up one flight of stairs at the application level, and will also increase the readability of the whole manuscript. "*

**[Authors’ response]:** Thank you very much for pointing out this important problem. We have made correction according to the reviewer’s comments. The relevant contents are provided below as a screen dump for your quick reference and we hope it can meet your request.

At last, the authors would like to give many thanks to reviewer 2’s constructive comments for improving the quality of this paper.

********************************************************

- *Reviewer: 3*

*Comments for the authors:*

1. *" A discussion section should be added after results to elaborate the link of your findings with the prior literature. "*

**[Authors’ response]:** Thanks for your great suggestion on improving the accessibility of our manuscript. It is the important deficiencies in this paper, and we have added the discussion section according to your good comments. The relevant contents are provided below as a screen dump for your quick reference and we hope it can meet your request.

At last, the authors would like to give many thanks to reviewer 3’s constructive comments for improving the quality of this paper.

********************************************************

*Academic editor*

*Comments for the authors:*

1. *" If applicable, we recommend that you deposit your laboratory protocols in protocols.io to enhance the reproducibility of your results. Protocols.io assigns your protocol its own identifier (DOI) so that it can be cited independently in the future."*

**[Authors’ response]:** We are very sorry for our negligence. We have followed your suggestions to deposit our laboratory protocols in protocols.io. The corresponding link is [dx.doi.org/10.17504/protocols.io.b4duqs6w](https://dx.doi.org/10.17504/protocols.io.b4duqs6w) and has been outlined in the Methods section.

2. *"Please ensure that your manuscript meets PLOS ONE's style requirements, including those for file naming."*

**[Authors’ response]:** Thank you very much for reminding us. We have followed your suggestions to check our manuscript and we think it has meet PLOS ONE’s style requirements.

3. *" PLOS requires an ORCID iD for the corresponding author in Editorial Manager on papers submitted after December 6th, 2016. Please ensure that you have an ORCID iD and that it is validated in Editorial Manager."*

**[Authors’ response]:** We are very sorry for our negligence of missing the ORCID. We have followed your suggestions to update my ORCID information in the editorial manager.

4. *" We note that you have provided additional information within the Acknowledgements Section that is not currently declared in your Funding Statement. Please note that funding information should not appear in the Acknowledgments section or other areas of your manuscript. We will only publish funding information present in the Funding Statement section of the online submission form. "*

[Authors’ response]: Thank you very much for pointing out these problems. We have removed the funding-related text from the original manuscript and update our Funding Statement in the cover letter.

5. *" In your Data Availability statement, you have not specified where the minimal data set underlying the results described in your manuscript can be found. "*

*" We note that you have stated that you will provide repository information for your data at acceptance. Should your manuscript be accepted for publication, we will hold it until you provide the relevant accession numbers or DOIs necessary to access your data. If you wish to make changes to your Data Availability statement, please describe these changes in your cover letter and we will update your Data Availability statement to reflect the information you provide."*

[Authors’ response]: Thank you very much for pointing out these problems. We have uploaded our experiment datasets to the Zenodo platform and obtained an unique DOI (<https://doi.org/10.5281/zenodo.5914059>). We also update our Data Availability statement in our cover letter.

6. *" Please state what role the funders took in the study. "*

[Authors’ response]: We are very sorry for our negligence of the statement of the funders’ role. We have include this amended Role of Funder statement in our cover letter.

At last, the authors would like to give many thanks to academic editor’s constructive comments for improving the quality of this paper
